# Improving care interactions (and training) in nursing homes with artificial intelligence

**DOI:** 10.1007/s11357-025-01557-1

**Published:** 2025-02-25

**Authors:** Marie Lefelle, Mouny Samy Modeliar

**Affiliations:** 1 ATILF, UMR 7118, Lorraine University, Nancy, 54000 Grand Est France; 2 CRIL Laboratory, UMR 8188, Lens, 62500 Hauts-de-France France

**Keywords:** Nursing homes, Artificial intelligence, Decision trees, Restricted Boltzmann machine, Causal forest, Care

## Abstract

As the population continues to age, nursing homes will increasingly play a key role in caring for dependent individuals. To enhance the well-being of the elderly, it is crucial to focus on the language skills used during care interactions. However, issues such as the taboo surrounding dependency, scandals involving private nursing home management, the pressure for caregiver efficiency, and the variety of care contexts make monitoring these skills challenging. One way to address this is by collecting in situ data, supervised by language researchers and caregivers specialized in elderly care. This is the approach we have followed: the data collected was then analyzed using machine learning models to provide caregivers with crucial insights for improving care outcomes. Our research highlights the importance of specific factors in language-based interactions, especially in varied care situations. Notably, we emphasize the careful use of humor and the impact of caregiver experience on the success of care sessions. Consequently, we advocate for caregiver training that is grounded in real-life practice, focusing on context adaptation, active listening, and dialogue with residents.

## Introduction

This research focuses on dependent elderly individuals and aims to contribute to the global effort to enhance the well-being and safety of nursing home residents, particularly in the context of Alzheimer-type pathologies and the optimization of care practices.

While numerous initiatives and research projects are already addressing this issue, many rely on data processed using artificial intelligence (AI) methods. For instance, OSO-AI, a start-up, has developed a device equipped with microphones that raises an alert when it detects concerning sounds. Another initiative, the Emobot robot, monitors the mental and emotional health of elderly individuals by analyzing facial expressions and vocal cues to detect mood changes [3]. Although these autonomous monitoring devices are relatively expensive, they provide essential information about the elderly to medical staff, caregivers, and even families. They can alert caregivers to events such as falls or distress and track mood disorders, such as depression or anxiety, especially when the elderly are alone. However, the primary focus of these devices is on the general environment of the elderly, rather than on their language or specific interactions. Their primary goal is to facilitate monitoring by transmitting alerts when necessary, underscoring the growing role of AI in elderly care [55].

Nevertheless, it is important to note that these devices often overlook the critical aspect of caregiver-elderly interactions. They primarily monitor the elderly person, without taking into account the dynamics of communication between the caregiver and the resident. Similarly, social robots, such as the Zora robot [4], popular in France, offer companionship by providing emotional support and cognitive stimulation, but they do not interfere with the relationship between the caregiver and the patient. In other words, very little research has been conducted on how caregivers manage communication during care, which is a potential area for improving the quality of care.

In France, the care of dependent elderly individuals in nursing homes is primarily handled by two types of professionals: paramedical staff, including nurses and care assistants, and social and medico-social professionals, such as “Accompagnants Éducatifs et Sociaux” (AES). The AES diploma prepares caregivers—mostly women, as the profession is highly feminized [14] to care for various dependent populations. Their role is to alleviate different forms of dependency, whether related to cognitive and linguistic development in children, disabilities, or age-related frailties. The diversity of their responsibilities is reflected in the wide range of workplaces, such as centers for children who have experienced trauma, facilities for disabled adults, and nursing homes for the elderly with multiple pathologies. The specific characteristics of each care setting influence the nature of interactions that occur there.

The French care system for dependent elderly individuals faces significant challenges, including shortages of time and personnel. In this context, AI tools are seen as valuable for supporting caregivers while also addressing the need for efficiency. Additionally, the rising number of individuals with neurodegenerative diseases like Alzheimer’s—affecting 40% of those admitted to nursing homes in France by 2019 [2]—makes AI-driven research into early detection [8, 24, 51] and care optimization even more crucial. These disorders introduce unpredictability and risk into care, which can be mitigated by attentive environmental monitoring [28], robotic assistants offering emotional support and cognitive stimulation [49, 58, 76], and better management of caregiver-patient communication, which could be enhanced by AI tools.

This paper addresses an often overlooked and underappreciated aspect of caregiving: language and communication. Our research takes an original approach by combining AI with the professional expertise of caregivers. While most existing methodologies focus on technical aspects of natural language processing (NLP), such as word lists and vectorization, our AI-based approach seeks to understand caregiver-resident interactions by analyzing both intrinsic and extrinsic parameters. Intrinsic parameters include phenomena related to the language used, while extrinsic parameters pertain to the broader caregiving environment. These factors encompass resident pathologies, levels of dependency, care settings, emotional states, and the training and experience of caregivers.

It is crucial to acknowledge the wide range of situations that can arise in a nursing home. For example, assisting a resident with Alzheimer’s and language disorders during mealtime requires a different approach than helping a coherent resident get out of bed. Moreover, caregiver competencies vary, so it is essential to identify the specific language skills that contribute to high-quality interactions. To address this, we adopted a two-stage process for data collection, which will be explained in detail in the “Methods” section.

Our research is part of a strong commitment to utilizing AI in the management of elderly care, particularly by acknowledging the profound impact of neurodegenerative diseases on aging populations. Our contribution introduces an original AI-based approach that focuses on enhancing the interaction between caregivers and residents, with the goal of improving caregiver training and, ultimately, the quality of care provided. Unlike local devices-such as sensors, boxes, or robots typically installed in residents’ rooms-which often require significant financial investment that many care facilities cannot afford, our research offers general knowledge that will be made directly accessible to caregivers. This includes the ongoing development of a mobile application, available as free software.

However, this progress comes with its own challenges. The drive to innovate with tools that assist caregivers and detect illnesses must not overshadow the importance of the care relationship itself, which remains fundamental. In addition to data collection and AI-driven analysis, our aim is to provide a decision support application that empowers caregivers to improve the quality of interactions and care in nursing homes.

## Methods

In close collaboration with numerous caregivers, we chose to focus our study on the expertise of caregivers rather than relying on discourse analyzed or produced automatically by AI models, such as large language models (LLMs).

Indeed, even though progress has been made in learning the language of caregivers [56], we believe that some in-depth studies are necessary to fine-tune the training of LLMs. Additionally, in the procedure we used for collecting data, the caregiver is central in assessing the care sessions while taking into account verbal, nonverbal, and contextual elements, which would be very challenging for current LLMs. In other words, by centering our analysis on the nuanced understanding of caregivers, we aim to capture the complexity of caregiver communication, which may not currently be fully appreciated or accurately interpreted by automated systems alone.

While LLMs offer promising potential in generating interaction strategies, the unique complexity of caregiver communication requires a deeper and more human understanding. A framework provided by caregivers is essential for fully understanding these interactions. Only a caregiver, through her experience, can recognize the intention to reassure a resident in countless subtle ways that cannot be captured by predefined rules or algorithms. These complex skills, shaped by the caregiver’s deep contextual understanding, are crucial for interpreting and effectively responding to care situations, which even advanced AI models such as LLMs may currently struggle to reproduce.

### Data collection

To begin our research on interactions with elderly individuals in nursing homes, we first needed to collect data related to caregiving situations. This task proved challenging, particularly given recent scandals that have been widely publicized in the media. Access to healthcare facilities is often restricted for external observers. Consequently, we recognize that our data, collected from a single establishment, may carry biases and may not fully reflect broader trends across the sector.

The two types of data used in our analysis are logically and temporally connected. The first dataset, collected in 2019, served to identify key variables that informed the structure of our database, which was subsequently populated with data gathered in 2023.

Data collection took place at EHPAD Stéphane Kubiak in Oignies, a nursing home operated by the French group ‘La Vie Active’. This EHPAD has around 100 residents, 38 of whom are in a special Alzheimer’s unit. This EHPAD corresponds to the national average for the number of people with Alzheimer’s in retirement homes, which is 40% according to the “Direction de la Recherche, des Études, de l’Évaluation et des Statistiques” or French Directorate of Research, Studies, Evaluation and Statistics [2]. At the time of the two data collection steps, the average age of residents was over 80, which corresponds to the national average for people accommodated in these facilities [2]. While most of the residents are female, as is the case in the majority of nursing homes in France [2], other sensitive data, such as ethnic origin, are not accessible due to personal data protection regulations, such as the “Règlement Général sur la Protection des Données” or General Data Protection Regulation (RGPD) in Europe.

The 2019 corpus consists of 34 fully transcribed audio and video recordings capturing a broad range of interactions between caregivers and residents. These recordings highlight a diverse set of caregiving activities, such as assisting with meals, helping residents out of bed, and preparing them for sleep, with each scenario involving a different combination of caregivers and residents. The corpus showcases a remarkable variety of care situations, featuring around 40 distinct resident profiles, ranging from individuals with Alzheimer-type conditions to those with varying levels of dependency. This variety extends to the caregivers as well, ensuring a rich representation of different approaches and interactions in the caregiving process.

**Table 1:** Icon convention

Icon	Significance
	Caregiver
	Second caregiver
	Caregiver not identified in transcription
	Resident
	Second resident
	Student
	Teacher

**Table 2:** Transcription convention

Symbol	Significance
(.)	Pause
(..)	Long pause
(comment)	Indication about an unintelligible part
((action))	Description of an action
<((action)) sentence >	Description applied to the whole sentence (between < and >)

In the remainder of this paper, we will analyze these interactions, which take place during what we refer to as care sessions, care situations, care interventions, or care acts, depending on the context, and were collected within the nursing home setting. To cite specific interaction items, we will employ ICOR-like [5] conventions as outlined in Tables 1 and 2. Note that, for the sake of readability, we have preferred to use icons rather than identifiers, and to add a few punctuation marks when inserting the transcription extracts. This allows us to highlight actions and comments, which clarifies and contextualizes the discourse.

#### Key features of language-based interactions

Through contextualized transcription, we were able to conceptualize the lexicon as a set of features. In the context of elderly care in nursing homes, each feature represents a specific criterion, identified through a combination of technical and medical literature, including informal geriatric care guidelines, and extensive discussions with caregivers. This approach was necessary due to the lack of formal training in elderly care, as highlighted in reports such as the 2022 Cour des comptes analysis on medical care in nursing homes [20]

The first step in our approach is to identify the features, understood as influencing factors, that are most likely to affect interactions. Some of these features are linked to the resident, while others pertain to the caregiver. Understanding these features helps anticipate whether a resident’s response will be appropriate or inappropriate, thus enabling caregivers to better tailor their interactions. This approach forms the basis of the AI-driven method we introduce and explain in detail later in the paper.

In this section, we identify the key features that characterize the fundamental concepts of language-based interactions between caregivers and nursing home residents, according to three main dimensions:

- Care context
- Infantilizing language
- Language disorders

Among the variables most obviously considered influencing factors and likely to have an impact on interactions are those linked to the context of care like pain or aggression. Those situations can be transversal to all care situations regardless of the age of the patient/resident. For instance, the discomfort, such as pain, expressed by a resident could be observed in the corpus as follows: 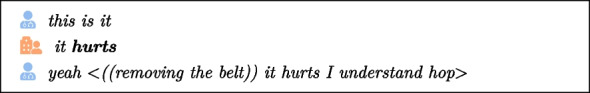 and 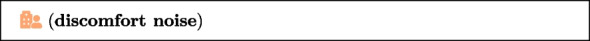 and 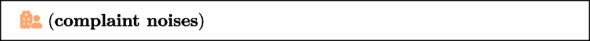


Any kind of aggression (verbal or physical) requires the careful involvement of the caregiver. This is illustrated below in the context of moving an elderly person from one room to another (warning: this extract contains coarse language which may shock but reflects the reality of care practice): 
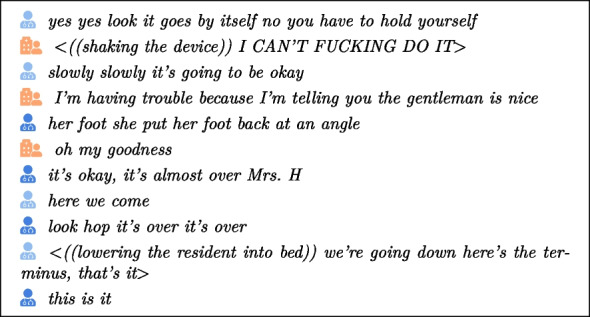


These factors will provoke a response on the part of the caregiver: familiarity [63], closed-ended questions [1], or repetition for example. We will illustrate this caregiver reaction with two additional examples. First, we begin with the concept of “renarcissism” [60], which focuses on helping individuals regain their self-confidence. This is reflected in the audio and video corpus through comments such as follows: 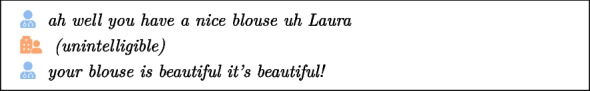


The second example highlights reassurance, demonstrated in extracts where caregivers provide comfort to those in their care: 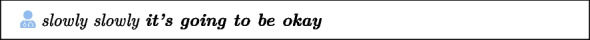 and 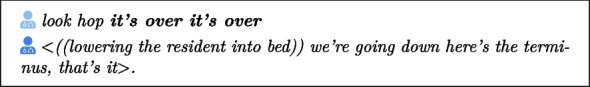 and 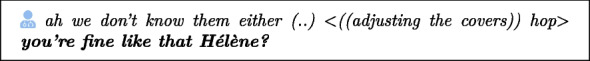 and 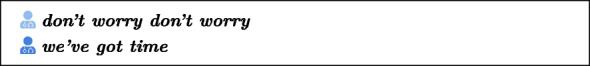


However, some interaction parameters are specific to the care of the elderly, such as the use of elderspeak.

We focus on the infantilizing language that may be used rather frequently in nursing homes and its potential impact on residents. Some parallels have already been drawn between a public made up of children and one made up of elderly people [71]. In particular, elderly persons are often infantilized by the use of what we call patronizing speech, over-accommodation, baby talk, or elderspeak. This form of language interaction results in a slower rate of speech [68], a form of familiarity and closeness^1^ [66], excessive repetition [68], exaggerated intonation [71], the use of collective pronouns (we, us) [67], and a limited vocabulary [39]. According to many studies, using elderspeak with older people does not improve care, especially for those suffering from dementia.

On the contrary, it causes resistiveness [80]. Consequently, the misplacement of a type of communication (initially intended for children) to an inappropriate target (the elderly) does have an impact on care interventions.

Moreover, caregivers should be aware that the aim of verbal interaction is not the same for children and elderly people. In the context of a dialog with the elderly, it is well known that a crucial issue is to slow down cognitive decline [29], by recalling elements that have been lost by the elderly person, as illustrated in this authentic extract from our corpus: 
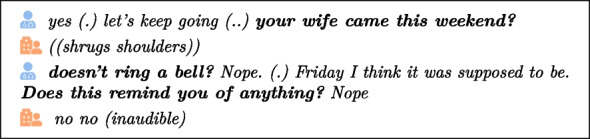


Here, a caregiver mentions a visit from the resident’s wife (your wife came this weekend?) even though the resident does not seem to remember. Then, he insists twice.

Following on from the remarks above, one can emphasize the differences when interacting with an audience composed of either children or elderly people. Indeed, when interacting with a child or toddler, the aim is to solicit the cognitive and linguistic development [18, 26, 61], whereas, with the elderly people, the aim is to preserve life-long acquired knowledge. Actually, for elderly people, it is essential to recall the basic concepts of everyday life, in particular when the person suffers from cognitive disorders. For example, in the following extract, the caregiver reminds the resident of her ability to eat on her own: 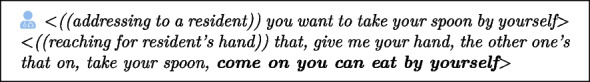


We believe that language disorders, particularly those stemming from cognitive impairments, must be taken seriously (consider the various pathologies that can affect the elderly). Alzheimer’s disease, which affects 40% of residents, is one of the most common illnesses in nursing homes in France [2]. This progressive neurodegenerative disease is often accompanied by language disorders, including perseveration [57], which refers to the inappropriate repetition of a verbal or gestural response. This can be observed in the following extract where the first syllable of the word “serious” is repeated by the resident, and then by the caregiver. This behavior stresses the resident’s difficulty in expressing herself and seems to lead to rejection.

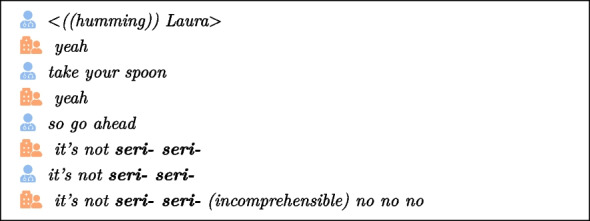



The various language disorders associated with this disease give rise to numerous unforeseen events in the interaction with the elderly person, such as aborted words [75] or a change in tone [16]. Another illustration is given below, where shouted words are written in capital letters (and preceded by “shouting” between brackets) and repetitions concern the words “no” and “go”: 
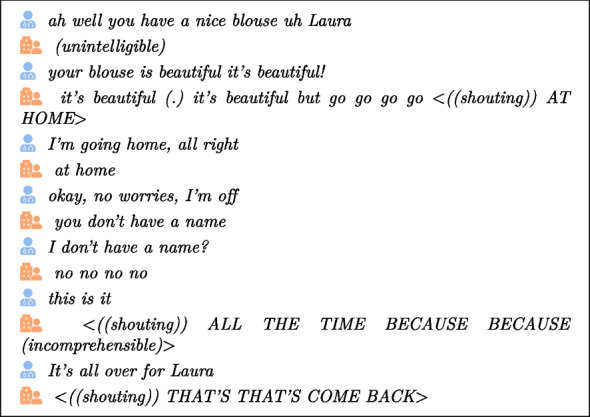


Identifying these features serves two functions. First, it highlights the importance of language skills in specialized settings for professionalizing practices. In the field of personal assistance, there is often confusion between intuitive help and professionalized help, leading to the misconception that assistance is accessible to all. However, this is far from true. The argument here is that caregivers can improve their communication in response to events by drawing on their experience and knowledge of best practices. Second, translating the lexicon into data science features enables the use of AI tools. These tools, especially in unsupervised machine learning, help identify the underlying causes of specific situations.

**Fig. 1:**
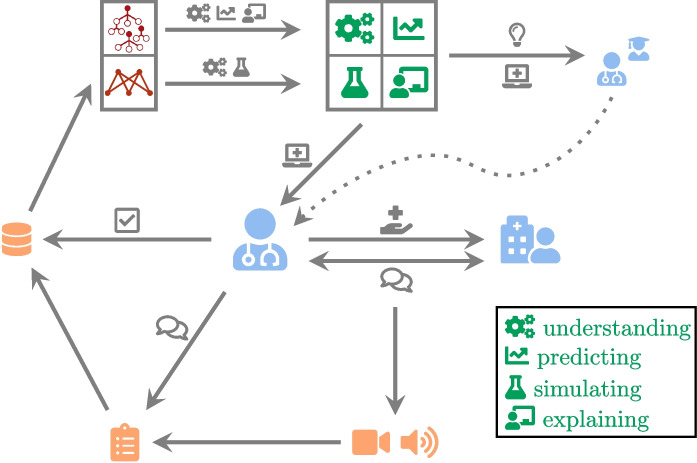
Human-centered analysis of care interactions. The caregiver is at the center of our study; their interactions initiate our reflection, and their experience allows us to adjust data collection and refine our models. The results of our research support the training and interactions of caregivers with residents

The method used in this study can be summarized in Fig. 1.

To summarize, our contributions are as follows:

- An *interdisciplinary collaborative work* carried out (over a period of three years) by people from both the Health sector (caregivers) and the Academic sector (researchers in Linguistics and Computer Science).
- An *extensive campaign conducted for collecting data* (audio/video recording of care sessions, questionnaire completed by caregivers) in nursing homes.
- The *identification of key features of language-based care interactions*.
- The *development of AI models* (random forest, restricted Boltzmann machine) for better understanding, predicting, simulating, and explaining care interactions.
- The *practical exploitation* (general knowledge of the importance of some features, and, forthcoming, a mobile application) of these models.
- *The demonstration that new challenges lie ahead for training*: Caregiver training should include in the future a strong focus on the language skills mobilized during care interactions with elderly residents.

#### The database

The database was created in August 2023 based on the analysis of the corpus. A teacher responsible for training future caregivers and assistance professionals conducted this second round of data collection. Ideally, the labeling process would involve multiple experts to cross-check and validate classifications, reducing personal bias and ensuring a more robust and reliable dataset. However, the teacher’s specialized expertise lends significant credibility to the task. Since the labeling was performed by an educator who trains future caregivers, this expertise helps mitigate bias and enhances the accuracy of the process. His pedagogical experience and in-depth knowledge of best practices in elderly care provide a solid foundation for precise and informed labeling, further improving the reliability of the data. It is also important to note that data collection should not be conducted by a caregiver unfamiliar with this population, which adds the challenge of finding professionals with the appropriate profile for such tasks.

This data collection was funded by a research prize awarded at the end of 2022^2^ and a research quality bonus obtained between our two research laboratories^3^. These resources enabled the comprehensive analysis of the resident-caregiver interactions within the facility.

These residents often present unique challenges, requiring caregivers to have specific training and understanding of neurodegenerative conditions.

Gender distribution is another important sociological factor to consider. As noted, most residents are female, which is consistent with broader trends in nursing homes across France, where women are more likely to live in these facilities than men [2]. This can be attributed to women’s longer life expectancy compared to men, meaning they are more likely to reach advanced ages where institutional care becomes necessary.

Regarding the data collection process, the use of video and audio examples to build an initial corpus was essential in identifying relevant features. The questionnaire, which was accessible via a tablet, allowed for the systematic observation of more than 503 interactions over a month. These interactions, involving around 100 residents and 50 caregivers, were observed across all sectors of the nursing home, including the Alzheimer’s unit. This sample size is substantial for the research methods used and provides a robust dataset for analyzing interactions in a caregiving context.

In this sense, the facility not only reflects national trends in aging and care but also serves as a microcosm of the broader dynamics within French nursing homes, offering insight into how care is delivered in environments that are increasingly shaped by the needs of an aging population. The data collected can provide valuable insights into the ways residents and caregivers interact, potentially influencing future care practices.

To delve deeper into these interactions, it is important to consider both the intrinsic features within the interactions themselves and the external factors that may shape them. For instance, the caregiver’s initial training, specialized courses on elderly care, and overall experience are likely to have a significant impact on the nature and quality of these interactions.

This section (background) of the questionnaire pertains to the aforementioned aspects: 
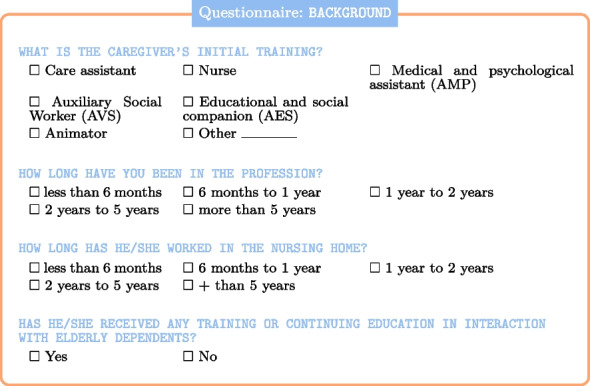


Particular attention has also been paid to various contextual factors, including the type of unit or service, the time of day, the type of assistance or care required, and the location. 
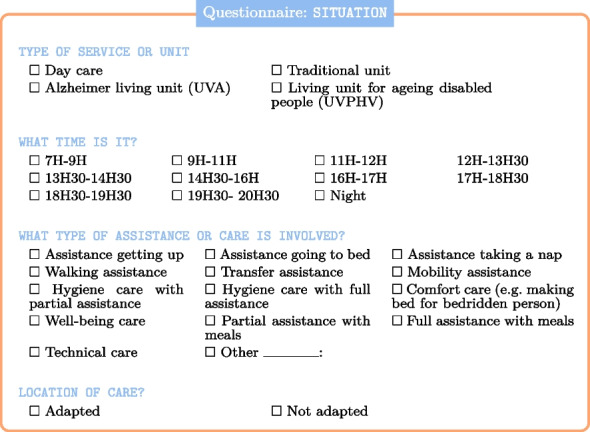


Also, in relation to the context, a caregiver who is accustomed to caring for a specific individual will have a different relationship with that person. This will have a clear impact on the interaction and care.

Unfortunately, nursing homes often experience high staff turnover [50], which can reduce the quality of care provided to residents [19].

Similarly, the degree of dependency or a pathology may influence the interactions. For instance, neurodegenerative diseases such as Alzheimer’s can make interactions (almost) impossible. 
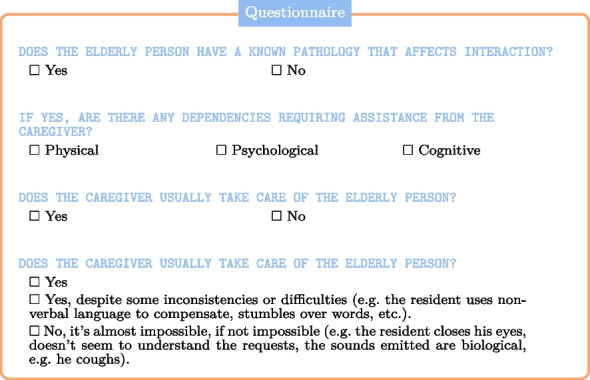


Finally, we have included the intrinsic parameters (variables) that are specific to the interactions. This includes triggers for the elderly person^4^ and reactionary gestures for the caregiver.^5^

This also includes factors that could be seen as infantilizing, such as using inappropriate vocabulary^6^ [42],

or depersonalizing such as substituting collective pronouns (for example, *we are not happy this morning* [9, 71]). 
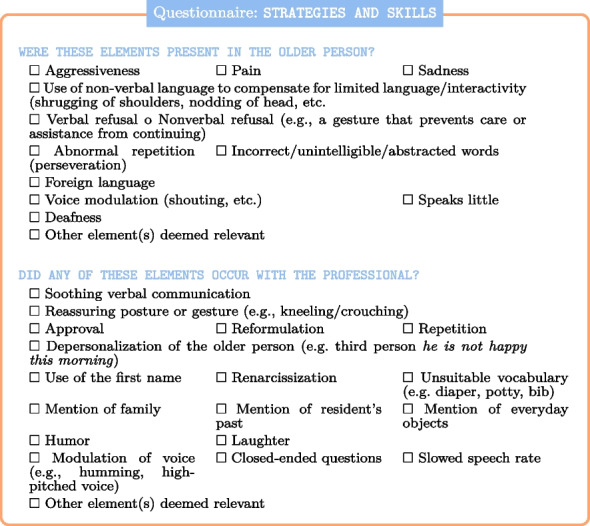


The final variable in the questionnaire relates to the success of the care, which can be classified as successful, partially successful, or unsuccessful. Caregiver interpretation of real-life interactions is crucial, as the quality of the data directly impacts the effectiveness of training an AI model. This is why, during data collection, we prioritized the caregiver’s expertise and minimized reliance on automatic interpretation. Furthermore, the results of trained AI models must be interpreted by humans. This interpretation is ultimately entrusted to a human specialist, which inevitably introduces a degree of subjectivity. 
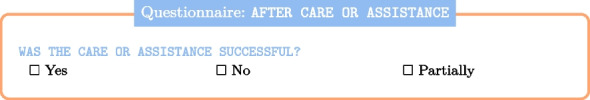


#### The dataset

The final stage involved transforming the three non-binary responses—regarding treatment success, the caregiver’s overall experience, and their experience in nursing homes—into binary variables to serve as inputs for our AI model. This decision was driven by the efficiency of some of our models when applied to binary data. Additionally, we aimed to develop a mobile application for caregivers, which would not have been feasible without this transformation. This conversion required interpreting the three corresponding questions based on criteria derived from our discussions with caregivers. Regarding caregiver experience in nursing homes, we classified any experience of less than one year as “low.” For this question, the questionnaire includes the following elements: 
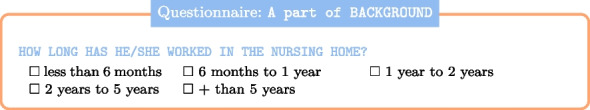


The corresponding binary variable for “solid experience” in this case has two possible values: 0 for “weak experience,” which applies to the first two answer choices, and 1 for “solid experience,” which applies to the remaining answers. Therefore, a caregiver with exactly one year of experience is categorized as “experienced,” though a different threshold could have classified them as inexperienced.

We chose to define a treatment as successful when it was fully completed. A completed treatment is coded with a value of 1, and 0 otherwise.

Moreover, while we opted for a binary approach, we acknowledge that different thresholds could have been applied, such as considering experience below 1.5 years as weak. Similarly, partial care completion could have been classified as successful. These alternative interpretations highlight the flexibility of the approach and suggest that multi-level encoding could be explored in future studies. However, for this initial stage, binary encoding provides a clear and effective starting point, especially given the subjective nature of some features like caregiver experience and care completion.

In the care sector, opinions on what constitutes adequate experience or successful care can vary significantly. Adjusting key thresholds, such as the minimum experience period or the degree of care completion, can lead to different interpretations of the same dataset. This flexibility might result in the creation of multiple datasets with varying binary variables. In this paper, however, we have limited our study to a single dataset. It is important to note that our discussions with caregivers strongly influenced the establishment of these key thresholds, giving us confidence in the relevance and applicability of our work. Moreover, we observed that all “reasonable” thresholds led to fairly similar results. Thus, we adjusted the experience threshold up to two years, and treatment success could include partial success. In all cases, the interpretations remained largely consistent. We selected the thresholds that most closely aligned with the caregivers’ perspectives.

This has resulted in 50 binary variables as follows:

**Table:** 

Training in Interaction PRO	Soothing Verbal Communication PRO
Reassuring Posture or Gesture PRO	Approval PRO
Reformulation PRO	Repetition PRO
Depersonalization PRO	Use of the First Name PRO
Renarcissization PRO	Unsuitable Vocabulary PRO
Mention of Family PRO	Mention of Residents Past PRO
Mention of Everyday Objects PRO	**Humor PRO**
Laughter PRO	Modulation of Voice PRO
Closed-ended Questions PRO	Slowed Speech Rate PRO
Aggressiveness RES	Pain RES
Sadness RES	Use of Non-Verbal RES
Verbal Refusal RES	Nonverbal Refusal RES
Abnormal Repetition RES	Incorrect Words RES
Foreign Language RES	Voice Modulation RES
Speaking Little RES	Deafness RES
Not Adapted Location	Pathology-Impacted Interaction
Usual Caregiver	Possible Interaction
**Successful Care**	Assistance for Getting up
Assistance for Going to Bed	Assistance for Taking a Nap
Assistance for Walking	Assistance for Transfer
Assistance for Mobility	Hygiene Care (partial assistance)
Hygiene Care (full assistance)	Comfort Care
Well-Being Care	Assistance (Partial) with Meals
Assistance (Full) with Meals	Technical Care
General Professional Experience	Nursing Home Experience

The terms RES and PRO are used to differentiate between features associated with residents and caregivers, respectively.

### Machine learning for care

The efficacy of machine learning techniques in the domain of medicine has been substantiated. In particular, they are capable of outperforming traditional statistical techniques. [73]. The application of AI in the field of medicine has yielded numerous successful outcomes over the course of its development [34, 38, 40, 43, 44, 62, 74, 74, 79]. There are numerous methodologies, and selecting the most appropriate ones for a given task is a crucial undertaking.

We can identify four purposes of using machine learning (ML) in the context of care interactions:

1. *Understanding* the importance, role, and correlation of variables, so as to better understand the interactions in nursing homes
2. *Predicting* the success of care sessions, which is equivalent to a classification task
3. *Simulating* various scenarios which can be seen as interactive decision support in the field
4. *Explaining* the reasons why or why not a care intervention was or was not successful

Although the first two purposes can be imagined mainly upstream, the last two are clearly relevant along the continuum of care. To achieve such objectives, we need to design relevant ML models and implement them in an application offered to caregivers. In this paper, we have chosen to base our work on two types of models.

The first is a decision tree model for its effectiveness on tabular data and for benefiting from recent works on explainability. We have specially chosen random forests (RFs) [17], which are composed of decision trees, because of their simplicity, readability, and ability to be explained. Learning such structures is relatively cheap compared to deep neural networks, and, according to [15], random forests are better at predicting tabular data than neural networks. The PyXAI explanation tool [12, 13] was selected due to its unique capability of providing comprehensive explanations for complex problems. Furthermore, the causal forest algorithm [10, 78] was employed to investigate a specific implication.

The second model is an energy model for its ability to interpret correlations and to make estimates in situations where information is incomplete. In particular, we use the restricted Boltzmann machine [33]. It is a good model for a mobile application for caregivers because it is very flexible.

#### Random forest

**Fig. 2:**
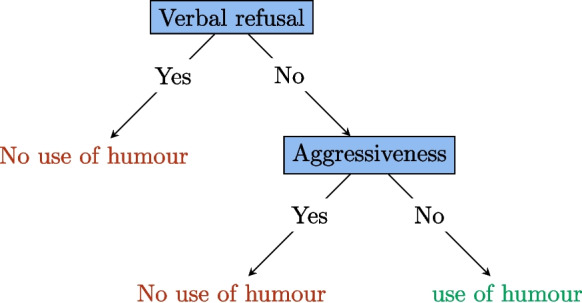
A binary decision tree. Each question is a node of the tree, and each answer is a branch. The terminal leaves of the tree represent the decisions to be made

A decision tree is a hierarchical decision support model that can be represented in the form of a tree whose nodes are attributes and whose leaves are decisions (as an example, see Fig. 2). In practical terms, one can follow the arcs of the tree, starting at the root node while answering encountered questions in order to arrive at a certain decision (which is related to a predetermined criterion). Figure 2 shows a simplified decision tree for a caregiver who wishes to carry out a course of treatment. The decision to be made is whether or not to use humor (which is the criterion of interest). To address this issue, two questions need to be asked. The first one is as follows: is the resident expressing a verbal refusal? If so, humor should not be used, and if not, the second question is as follows: is the resident expressing aggressiveness? If the resident is aggressive, humor should not be used; otherwise, it can be used.

**Fig. 3:**
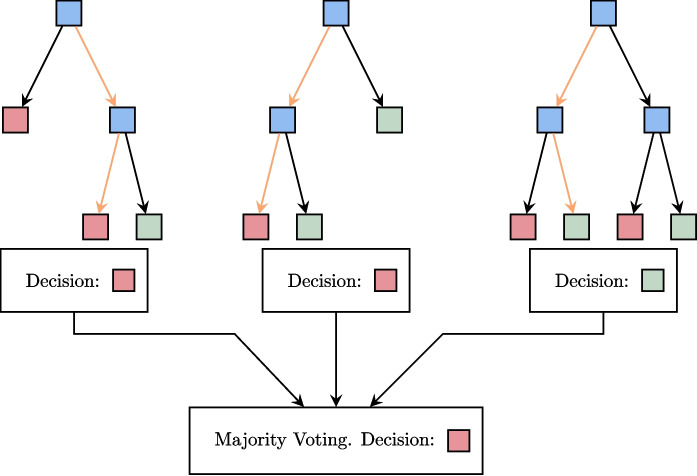
Random forests are composed of decision trees. The decision is reached through a majority vote, in which all proposals put forth by the trees are considered

Nevertheless, the construction of these decision trees is not satisfactory. Indeed, to start, a variable, representing the criterion of interest, must be isolated from the data to form the basis of the decision to be made. Then, such trees are constructed statistically from a dataset, and their shapes can vary depending on the specific algorithm used. Moreover, the data offers then statistical metrics such as frequency and entropy that can aid in selecting the nodes of interest in the decision tree. However, with homogeneous data, these metrics can be very similar, leading to significant decision variability. To control this variability, we generate a large number of datasets by randomly deleting elements from the original dataset. Each dataset then generates a decision tree. In the case of a binary decision, the majority of votes^7^ is used to make the final decision. This is the random forest (RF) algorithm. An illustration is given in Fig. 3.^8^

Note that among the parameters, called hyperparameters, of the model, we find the number of decision trees, their size, the objective, and the variables to be considered when building the trees.

**Fig. 4:**
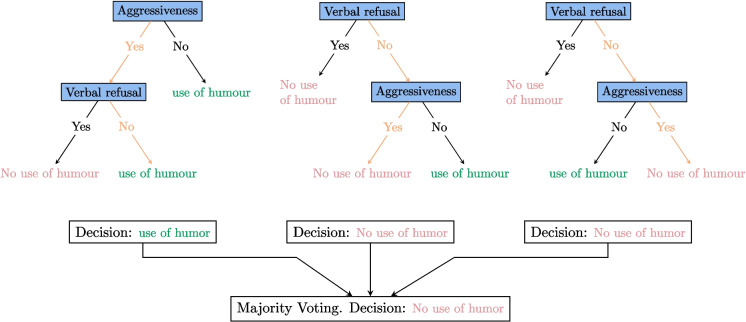
Random forests in real-life situations. There is aggressiveness but no verbal refusal. One tree advises using humor, while the other two suggest not doing so. The majority vote is not to use humor in this situation

Let us look again at the example in Fig. 2. We can imagine that this decision tree is built with a sufficiently homogeneous dataset that removing a few interactions will completely change the tree. So let us imagine that we use the random forest algorithm to generate 3 trees. The dataset then randomly generates 3 datasets which are subsets of the original dataset. These sets produce the trees shown in Fig. 4. In the event that the carer encounters a situation in which the resident does not verbally refuse the care but displays aggressive behavior, the final two trees will determine that the use of humor is not appropriate, in contrast to the first tree. The majority decision will then be to refrain from using humor in this situation. The benefit of this approach is that it yields more consistent and enduring decisions that do not necessitate the modification of all decisions when a resident arrives or leaves.

Rather naturally, we chose “Successful Care” as our target variable.

To evaluate our model with this target, we will use metrics such as *accuracy*, *precision*, *recall*, and *F1-score*. We can see this in terms of true positives (TP), true negatives (TN), false positives (FP), and false negatives (FN).

The definition of *accuracy*, which answers the question “overall, how often is the model correct?” This is the proportion of true among all the results (TP+FP).

The definition of *precision*, which answers the question “overall, when the model predicts positive, how often is it correct?” It is the proportion of true positives among all positive results. Hence, in our context, precision is useful to analyze predictions regarding the success of care acts.

However, the crucial factor is whether our model accurately detects positive results. To achieve this, we can utilize the *recall* metric. The definition of *recall*, which answers the question “overall, does the model correctly detect positives?” This is the proportion of true positives among the truly positive instances (TP+FN). In our context, recall can be seen as the ability to correctly identify successes.

Precision and recall are complementary measures, and they can be combined into a single measure called the *F1-score*. The definition of *F1-score*, which answers the question “overall, does the model correctly detect positives?” The F1-score is a harmonic average of the precision and recall metrics. A higher F1-score indicates a better model.

It is also possible to obtain measures to assess the feature importance. Each measure defines a particular feature importance. First, we use the following classical measures that were used to investigate the importance of RF features:

- Mean decrease in impurity (MDI), or Gini importance
- Mean decrease in accuracy (MDA), or permutation importance

The MDI metric extracts the characteristics that most discriminate the output, i.e., those that contribute the most to reducing the number of errors. The MDA metric is based on a procedure that randomly varies certain variables in order to measure their influence on the model’s accuracy.

It is interesting to compare the results obtained using these two methods in order to enhance the reliability of result interpretation.

MDI allows for the extraction of the most discriminative variables in the decision-making process, while MDA identifies the variables that contribute the most to the model’s accuracy.

In practical terms, the variables identified using the MDI/MDA methods are interpreted as key elements within the decision tree model, as they significantly influence the decisions made by the model. This means that, for caregivers, the features with the highest scores should be prioritized to facilitate quick and informed decision-making.

**Fig. 5:**
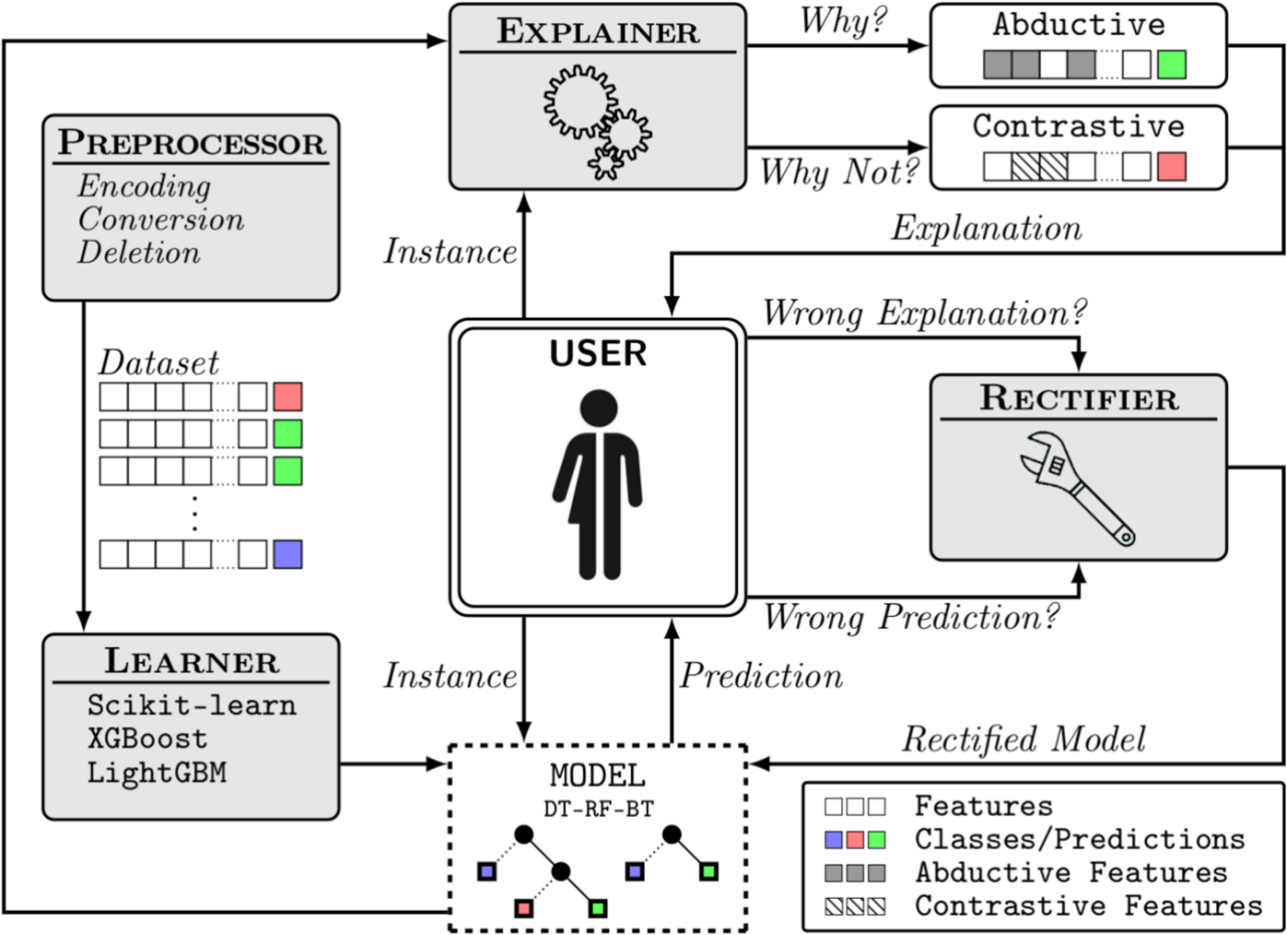
PyXAI (image source: https://www.cril.univ-artois.fr/pyxai/) is an explainability tool developed by the CRIL Computer Science Laboratory. It provides detailed explanations of the decisions made by machine learning models

For example, in the context of successful care outcomes, if variables such as “Humor,” “Verbal Refusal,” and “Aggressiveness” are identified as particularly important, the caregiver will recognize that these characteristics are strongly associated with the success of the care. However, it may not be immediately clear whether these variables have a positive or negative impact. The professional’s experience will play a crucial role in accurately interpreting these indicators. For instance, they may observe that humorous exchanges, the absence of verbal refusal, and low aggressiveness are generally associated with better care outcomes. Thus, at the beginning of a care session, the professional might evaluate these criteria to adjust their approach. For example, if they observe an absence of aggressiveness and verbal refusal, they may choose to use humor to maximize the chances of success.

However, this approach does not allow for the identification of whether certain criteria are essential for the successful care. Therefore, it is crucial to refine these analyses to better support caregivers. In this context, we also integrate the PyXAI method, which provides complementary insights into the relationships between variables and the conditions necessary for successful intervention.

#### Providing explanations with PyXAI

As we have seen, it is important to explain the reasons behind the practical results obtained with models. Our approach to post-care analysis then lies in the area of XAI (eXplainable AI). There are many popular approaches (SHAP, LIME,...) to XAI that are model-agnostic (see [23, 27, 35, 36, 46, 52, 59, 72] to cite a few). However, they usually do not offer any guarantee of rigor.

Correctness is paramount when dealing with high-risk or sensitive applications, which is why we decided to use the library called PyXAI [12, 13].

PyXAI algorithms rely on logic-based, model-precise approaches for computing explanations. Although formal explainability has a number of drawbacks, particularly in terms of the computational complexity of the logical reasoning required to derive explanations, steady progress has been made since its inception. Currently, with PyXAI, you can use methods to find explanations suited to different ML models for classification or regression tasks: decision tree (DT), random forest (RF), boosted tree (gradient boosting) (BT).

**Fig. 6:**
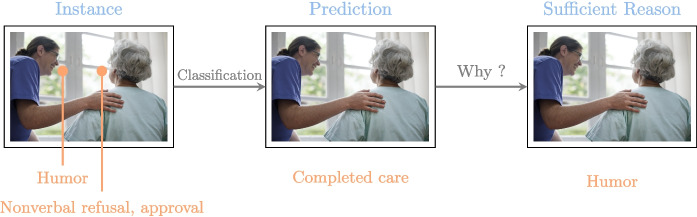
Explanation—sufficient reason. When applied to interactions, PyXAI highlights the essential features for the successful care

In our case, PyXAI will be useful to analyze the output of the RF model we used. We focused our study on care success, but any other output variable could have been considered.

For a given instance, PyXAI can give the reasons for classifying an instance in one class rather than another. These reasons are given in the form of conditions on the variables of the model. In the binary case, the explanation resembles a list of explanatory elements for certain variables, which may or may not be present.

Actually, there is a very large number of reasons that can explain the way a decision tree has been learned. PyXAI is able to extract minimal reasons by following the process depicted in Fig. 5. The minimal reasons can be of several types: (i) abductive explanations are intended to explain why an instance has been classified in the way it has been classified by the ML model (thus, addressing the “Why?” question), and (ii) contrastive explanations are intended to explain why the instance has not been classified by the ML model as the user expected it (thus, addressing the “Why not?” question).

These reasons are interesting because they can be used to determine what would need to be changed in order to change the predictive class.

To create the decision trees, we selected the xgboost algorithm from the PyXAI library. We used this method to extract the minimal reasons for the success or failure of a particular care session, so that we could analyze its strengths and weaknesses (as illustrated in Fig. 6). In this way, for a particular care, we are able to extract reasons that must be maintained if the care is successful and reasons that must be changed if the care is a failure.

What is important is that several minimal causes can explain a given instance. There can be a large number of them [11]. However, it is possible to calculate the number of minimal reasons to generate in order to get the most representative ones.

We then counted the number of occurrences of each variable (with its value) in these minimal reasons, in order to determine the variables that are most often involved. This cannot be interpreted globally (i.e., for all types of care sessions), but it does mean that the most common variables very often play a significant role in the outcome.

Some of these variables do not necessarily occur frequently in the care process, but as they tend to occur frequently in causing a failure, they should not be overlooked.

For example, in the context of seeking explanations for care success, the failure of a care intervention could be interpreted by PyXAI using the following vector:

$$\begin{aligned}&\text {(Pain RES: Yes, Reassuring Posture or Gesture PRO:}\\&\text {No, Technical Care: Yes).} \end{aligned}$$

This refers to a technical care procedure for a resident experiencing pain. While it may be difficult to directly alter the nature of the care itself, modifying the variable “Reassuring Posture or Gesture” shifts the outcome category, thereby turning the care into a success. PyXAI informs the caregiver that adopting a reassuring posture or gestures is likely to ensure the success of the procedure. The caregiver may also choose to address the pain management or delay the procedure until the pain subsides.

In other cases, PyXAI helps to identify key best practices to maintain. For instance, in the same context, if PyXAI provides the vector (Pain RES: Yes, Reassuring Posture or Gesture PRO: Yes, Technical Care: Yes), the caregiver understands that maintaining a reassuring attitude is a crucial factor for the success of the care.

Finally, PyXAI may also indicate a low probability of success in certain situations. For example, in the context of analyzing care success, if PyXAI provides the vector (Technical Care: Yes, Possible Interaction: No, Usual Caregiver: No), it is likely that only the usual caregiver would be able to successfully perform the intervention.

This approach thus enables the caregiver to identify key elements that may need adjustment, postpone the care if necessary, or seek a caregiver better suited to the context.

PyXAI suggests that in specific situations, modifying certain variables can influence the success of care interventions. This raises questions about broader implications, which will be explored in the following section through the use of the causal forest method. In this paper, we will focus specifically on the impact of humor on care success.

#### Causal forest

The causal forest algorithm [10, 78] is an extension of the random forest algorithm, with a particular focus on estimating causal effects. This algorithm aims to estimate the conditional average treatment effects (CATE). If this estimation is positive, it indicates that the treatment effect is considered significant. The measure *CATE* [37], the conditional mean treatment effect, is an estimator of the causal effect of one variable on another. It is calculated by estimating the effect of unit changes in one variable on another. In theory, to show that one variable has an effect on another, a change in the first variable must lead to a change in the second, while all other variables remain invariant.

In our study, we focus on the impact of humor on care success. Preliminary results show that humor plays an important role in the success of interventions. The application of the causal forest algorithm will help identify situations where humor contributes positively to care, as well as those where caregivers should avoid it. This will provide caregivers with precise guidance on the appropriate use of humor in their practice.

In this model, we will use the SHAP measure, which proves to be effective in this context. Similar to MDI and MDA, SHAP allows us to estimate the importance of variables. It is based on Shapley values [10, 78], derived from game theory, and provides a more detailed understanding of how each variable contributes to the model. We did not use this approach in previous studies as it produces results similar to MDI and MDA in terms of variable importance, and its explanatory power was less relevant to our context compared to PyXAI.

**Fig. 7:**
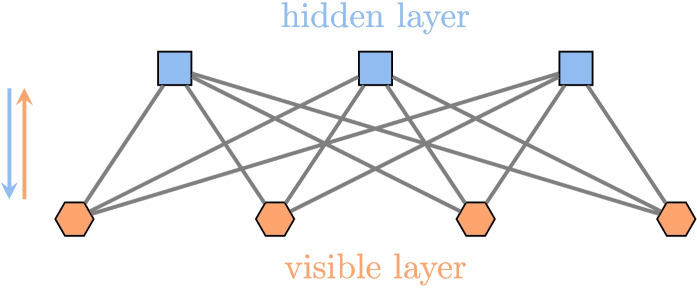
A restricted Boltzmann machine. It is a neural network for unsupervised learning capable of completing missing data

#### Restricted Boltzmann machine

If random forests are a useful tool for the purposes of “understanding” and “predicting” (as we have just seen in the earlier subsection), they appear to be less flexible for “simulating.” Simulating scenarios means, in particular, being able to deal with incomplete data and asking, for example, what is the expected outcome (success or failure) or how can the probability of success be optimized.

In practical terms, the mobile application currently under development, based on our research, integrates our models. The caregiver wishing to use this application during an intervention will need to input relevant care-related information. However, with 50 variables to complete, the caregiver may not always have the time or access to all necessary information. We therefore propose a flexible solution, allowing the caregiver to enter only the available or relevant data. The missing variables will be estimated using another neural network. This process will provide a probability for each variable, and the pre-existing random forest model can be enriched with these estimates to predict the likelihood of a successful care outcome. This estimate will also be used in PyXAI to advise the caregiver of the outcome. It is also worth noting that the binary nature of the variables simplifies the data entry process for the caregiver.

To deal with a wide variety of possible scenarios, one could possibly develop multiple random forests, each using one variable (or a limited number of variables) as the pivot for inference or reasoning.

However, this rather complex task is let as a perspective of this work. As a more immediate approach, we propose to use an RBM (restricted Boltzmann machine), which is both a simple and powerful tool to play with incomplete data and thus simulate scenarios. In addition, RBMs are an easy way to observe correlations between variables. To some extent, and at least in our context, RBMs can be seen as complementary to RFs.

A restricted Boltzmann machine is an unsupervised probabilistic learning model, which has been particularly studied by G. Hinton [6, 30–33, 41, 64, 65, 69, 70]. It has been notably used to predict energy consumption in smart cities [7, 21] and in the health sector [54]. An RBM allows the prediction, classification, and interpretation of the coefficients of its underlying structure, due to its theoretical Markov field model.

In our context, this model is adapted to the size and nature of our dataset.

Interestingly, an RBM can be used to make forecasts based on incomplete data.

The machine then provides an estimate for variables that cannot be determined.

Let us start with a brief explanation of how an RBM works.

Specifically, there are nodes (neurons) arranged in a manner as shown in Fig. 7. The network (graph) consists of two layers: the first (bottom) layer, known as the *visible layer*, corresponds to the data while the second (top) layer, known as the *hidden layer*, can be interpreted as categories associated with the data. The nodes in one layer are not connected to each other, but each node in that layer is connected to all the nodes in the other layer (forming a complete bipartite graph).

From an operational point of view, each node, seen as a neuron, in a layer receives input from all the nodes in the other layer.

As our data is based on binary variables, we will use a so-called Bernoulli-RBM, for which we can prove that the activation functions must be sigmoid [22].

**Fig. 8:**
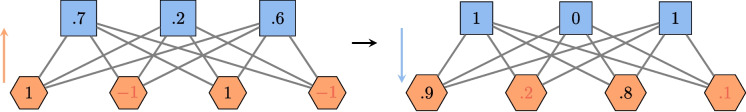
Reconstruction process. The RBM has a learning process based on the back-and-forth flow of information through neurons. This process, based on the Gibbs sampling method, allows for the completion of missing data

**Fig. 9:**
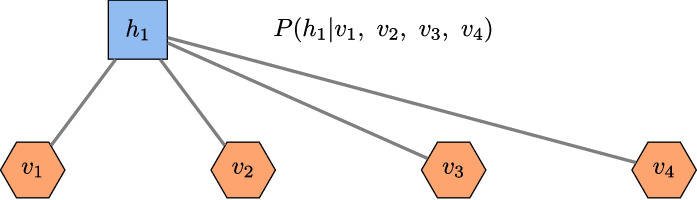
Conditional probability associated with hidden neurons. The coefficients connected to a hidden neuron can be interpreted as the probability of the neuron’s presence given the condition of the visible neurons being present

As in a conventional neural network, the edges of the graph correspond to the coefficients to be adjusted. If there are $n_{v}$ visible nodes and $n_{h}$ hidden nodes, the set of coefficients can be given by a matrix of dimension $n_{v} \times n_{h}$. The network propagates in two directions. The output values of the hidden nodes can be calculated from a common input.

These values, ranging from 0 to 1, can be simulated as Bernoulli random variables. In turn, they provide input to the visible nodes in the opposite direction.

This is known as *reconstruction*. The network is trained using data of the same size (number of neurons) as the visible layer by adapting Gibbs sampling [22, 30]. Once trained, the RBM can provide a lot of information.

Let us take a look at the visible layer.

Each component corresponds to a piece of binary information. Note that if some input values are unknown, they can be replaced with the value $-1$. This is because the energy function used to optimize the problem is in exponential form.

The initial propagation to the hidden neurons produces values that represent the activation probabilities of those nodes.

They are randomly simulated with this probability in a binary manner. The reverse propagation to the visible neurons allows a re-estimation of the nodes in the visible layer, which allows the evaluation of unknown variables.

The process can be repeated until convergence is reached, but in practice, a hyperparameter can be used to limit the propagation (in our case, it was set to 3).

An illustration is given in Fig. 8, with a network composed of 4 visible nodes (corresponding to the size of the data) and 3 hidden nodes (corresponding to the number of categories) following a hyperparameter of the model chosen according to the context. Here, we assume that this RBM is already trained. Regarding the input data, two of the four values are unknown: $-$1 is associated with the second and fourth neurons.

The values associated with the hidden layer are then calculated to be 0.7, 0.2, and 0.6 for the three hidden neurons.

The next step is a binomial simulation which provides, for each hidden neuron, a value of 0 or 1: 1 for the first and third neurons and 0 for the second one. Finally, the values associated with the neurons in the visible layer can be recalculated.

The value of the second neuron is estimated to be 0.2, which means that the probability that the value is 1 is 20%.

Similarly, the value of the fourth neuron is estimated at 0.1, which corresponds to a probability of 10% that this value is equal to 1. Although this process can continue, this is not shown here.

**Fig. 10:**
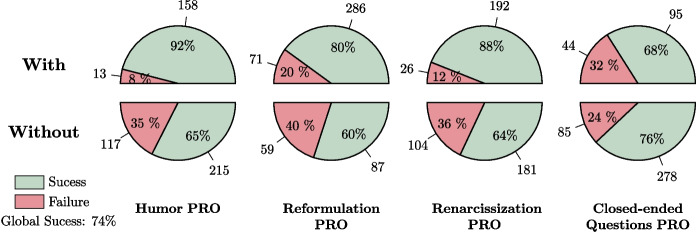
Strong impact of humor on successful care sessions. Here, we see the four most impactful factors. Humor appears to be a significant factor in success

During the reconstruction stage, an RBM may make errors and estimate a value that is the opposite to the one we know to be correct. The proportion of such errors is called *error per reconstruction* and serves as an initial metric for evaluating the model. When focusing on a single variable, it is also possible to make predictions on one or more attributes and calculate accuracy, precision, recall, and F1-score (see page 11).

Finally, apart from the five metrics mentioned above (error per reconstruction, accuracy, precision, recall, F1-score), it is worthwhile to note that each node in the hidden layer is useful for identifying some correlations between variables. Indeed, as can be seen in Fig. 9, any part of the network composed of one hidden neuron together with its neighbors (visible neurons) can be interpreted as a conditional probability. Thus, the hyperparameters can be interpreted as correlation indices between the neurons of the visible and hidden layers.

Technically, to accomplish this, we must evaluate the significance of the weight matrix coefficients on the sigmoid output.

In our mobile application, each neuron in the visible layer corresponds to the care features we have identified (“The dataset,” page 8). The caregiver enters information via their phone or tablet, assigning values to certain features in the visible layer. Upon validating the form, the reconstruction of the RBM (restricted Boltzmann machine) is triggered in the background. This process generates probabilities for the variables corresponding to the fields that were not completed. The caregiver thus receives an estimate of the probability of care success, along with an action plan to optimize the outcome. They can also modify or complete certain values to observe how the probability changes.

For example, a caregiver might indicate a technical care situation and sadness but no pain. The RBM will then estimate the remaining missing variables and inform the caregiver. Naturally, the more information the caregiver provides, the more reliable the estimates become. These values are subsequently used to determine the probability of care success, while PyXAI is employed to define an optimal care strategy. A system to prioritize the selection of variables to be entered in order to optimize care success is currently under development.

## Results

### Descriptive analysis

As a start, we decided to carry out a descriptive, coarse-grained analysis of our dataset in order to obtain some general and relevant information about the interactions. Based on the data collected, we first observed that 74% of the care sessions were successful while 26% were either incomplete or unsuccessful. This is quantified by the feature “Successful Care.”

However, certain features may have a greater or lesser impact on the success of care sessions, such as the use of humor in caregiving.

**Fig. 11:**
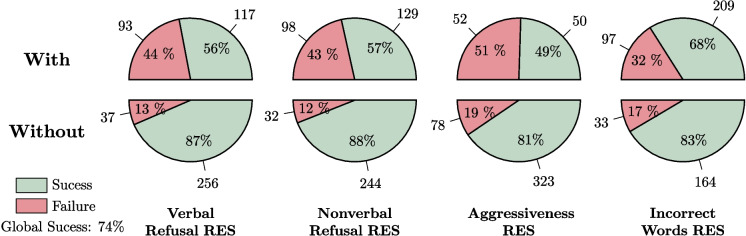
The four resident features that seem to have the greatest impact on the successful care

Figure 10 shows the four variables that appear to be most important for the success of care with respect to the caregivers’ actions. Figure 11 displays the four features that seem most significant for the success of care in relation to the residents’ reactions or states. As seen in Fig. 10, the success rate drops to 65% when the caregiver does not use humor during care sessions, but rises to 92% when humor is used. Only 13 care sessions failed when the caregiver used humor. It can also be observed that reformulation and renarcissization are slightly less important factors, yet still very significant. Closed-ended questions tend to hinder the success of care, although this strategy is generally associated with individuals who have cognitive impairments, which is not the case for the majority of residents in nursing homes. The use of closed-ended questions is intended to simplify responses, but it can sometimes be perceived as an infantilizing way of speaking, inappropriate for recipients who do not require it.

Figure 11 highlights the importance of the resident’s refusal in the success of care. For a relatively balanced distribution of cases between the two categories, care is 31% more likely to succeed when the resident does not refuse, either verbally or non-verbally. Additionally, considering the patient’s nervous state through aggressiveness and their physical state through the incorrect use of words are also important factors.

**Fig. 12:**
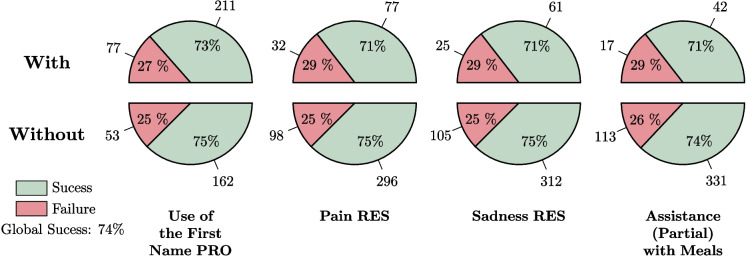
Four features that do not seem to impact the success of the treatment, contrary to what one might expect. In particular, we can observe the weak Impact of Using the First Name on Successful Care Sessions (only 2% improvement)

Finally, some variables appear to have no impact on the success of care. Figure 12 presents four variables that, counterintuitively, do not seem to have any significant effect.

As seen in Fig. 12, the success rate of care procedures is only marginally affected by the use of the patient’s first name. In fact, procedures are successful 75% of the time when the first name is used, compared to 73% when it is not used. The use of it is also balanced within the collected data, as it appears in 77+211=288 care sessions and is absent in 53+162=215 care sessions.

Interestingly, descriptive analysis enables us to make initial observations about the data. However, descriptive analysis alone cannot predict the contribution of binary variables to care, estimate unknown parameters, or explain connections between variables that contribute to successful care sessions. In particular, this analysis does not reveal the correlations or causalities between the variables.

This is why we propose an AI-based approach.

### Random forest

**Fig. 13:**
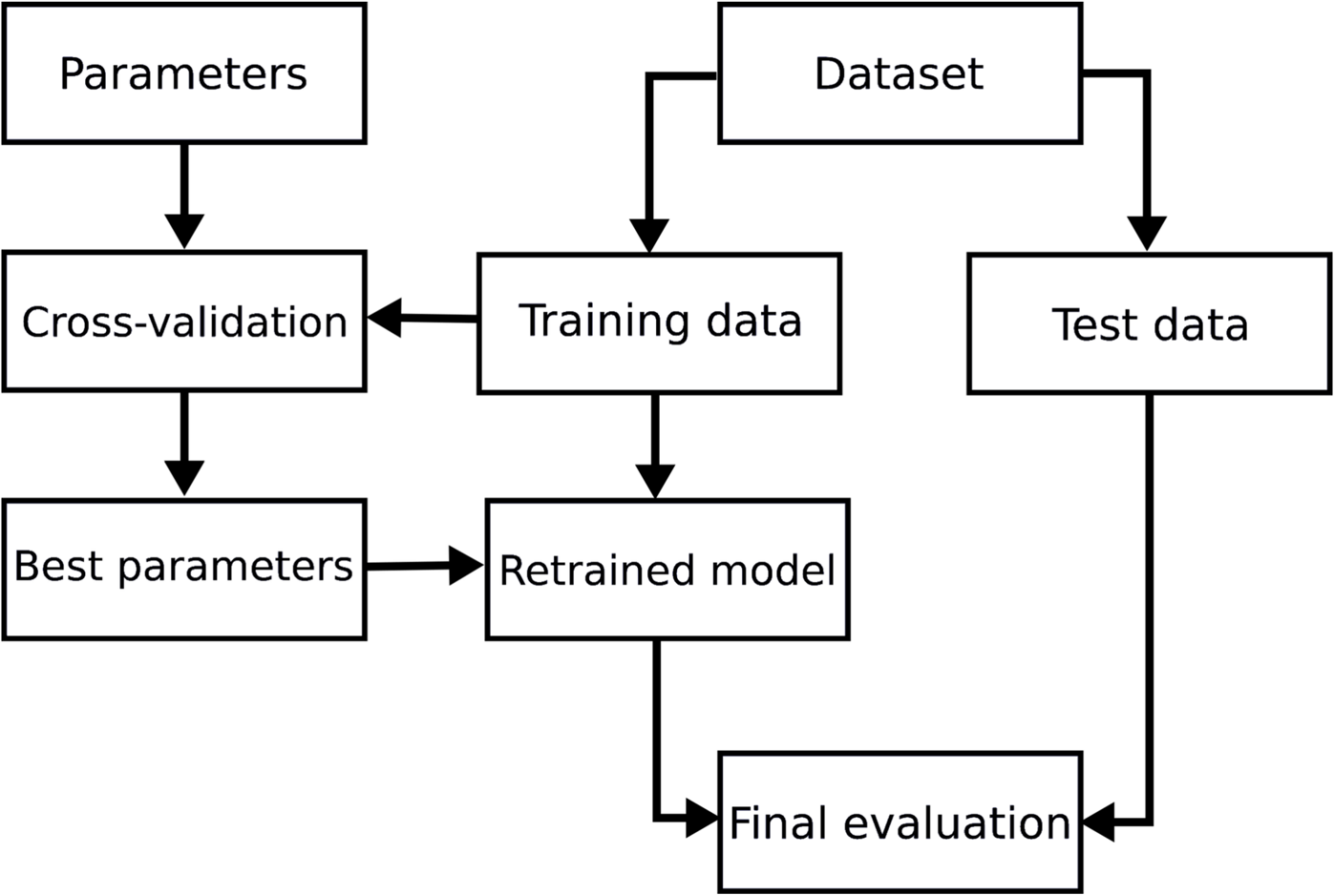
Diagram of parameter estimation and model training. Source scikit-learn

The random forest algorithm was applied with 500 decision trees, a minimum of 2 leaves per tree, and no maximum depth. The procedure used to obtain these parameters is summarized in Fig. 13.

The 503 interventions were randomly split, with 30% of the data used for testing and 70% for training, resulting in 151 interventions for testing and 352 for training. Cross-validation was performed, involving multiple training care sessions on datasets created from the training data to assess the quality of learning. The results are balanced and do not indicate overfitting. A grid and random search were conducted to optimize the model parameters. Finally, the model was trained on the training data using the obtained parameters, and tests were conducted on the test data.

The metrics used to evaluate the learning process are accuracy, precision, recall, and F1-score (see page 11). Table 3 presents the performance of the random forest model on both the training and test datasets. The most significant results are those on the test dataset, as the model did not have access to this data during training.

It is worth noting that using other reasonable thresholds to convert our data yielded similar results. The features that can significantly impact the results are few and are primarily related to the professionals’ experience. This leads to less than a 10% variation in the metrics and minor changes in the ranking of feature importance.

Table 3 shows a test set accuracy of 84%. This means that for the caregiver, our model correctly predicts the success or failure of the care session 84 out of 100 times.

Our model has a precision of 94.4%. Therefore, when the model predicts that the care session will be successful, the caregiver can be 94.4% confident that this prediction is correct. When the model predicts a care session failure, the reliability is 70% (not shown in the table).

Our model has a recall of 86.1%, correctly identifying the success of a care session in 86.1% of care sessions. For the caregiver, this means that few successful care sessions will go undetected by our model. The detection rate for care session failures is 84% (not shown in the table).

The F1-score combines both of the previous measures. The value of 90.1% indicates to the caregiver that the model not only correctly detects the success of a treatment but is also quite reliable when it does so.

**Table 3:** Results with the random forest approach

Scope	Accuracy	Precision	Recall	F1-score
Training	99.7%	100%	99.6%	99.8%
Test	84.3%	94.4%	86.1%	90.1%

The model performs very well at all levels according to the most common metrics. The line corresponding to the test data is the most significant

**Fig. 14:**
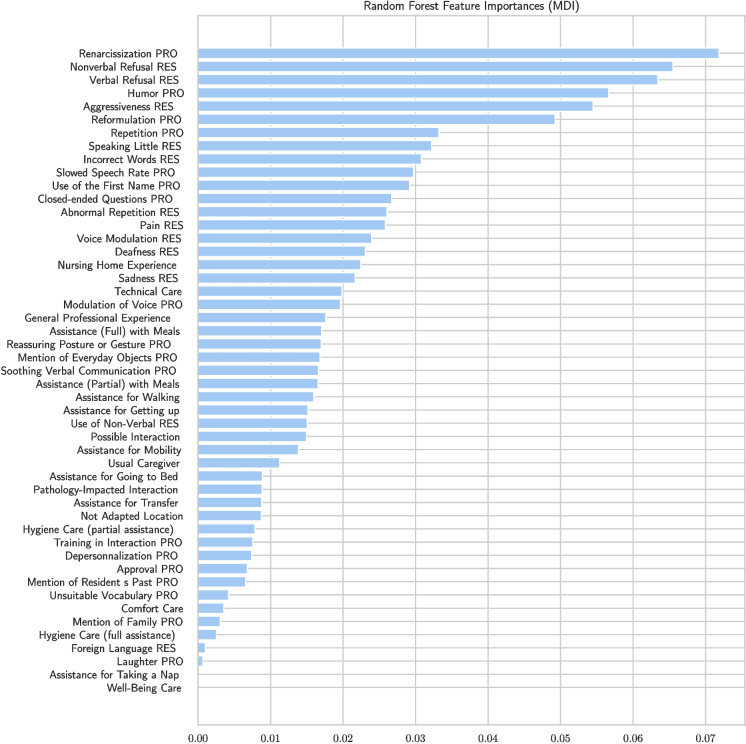
Importance of variables according to MDI. Here, we can see the features most useful to the caregiver’s decisions according to MDI

**Fig. 15:**
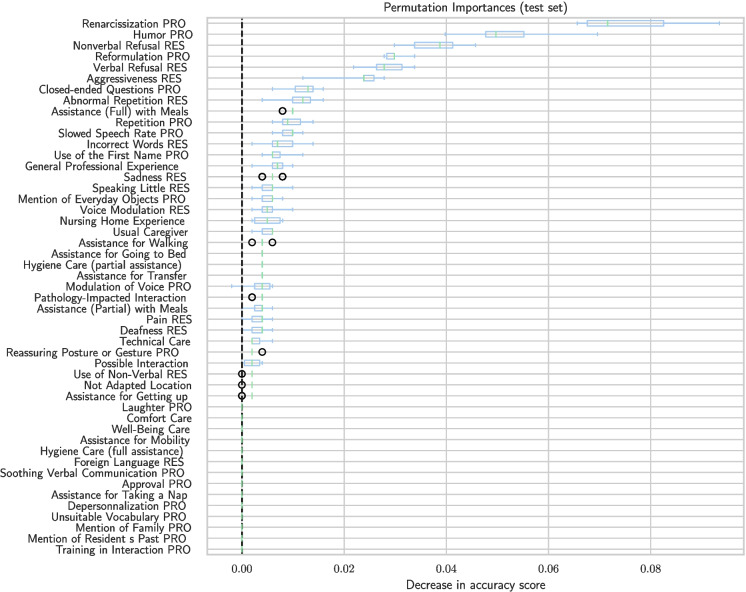
Importance of variables according to MDA. Here, we can see the features most useful to the caregiver’s decisions according to MDA

The relative importance of the features according to MDI and MDA measures is shown in Figs. 14 and 15, respectively.

According to the two measures, the features most often used to decide whether a treatment is successful or not are interaction-related features.

For the resident, the most decisive elements are verbal or nonverbal refusal, aggressiveness, and repetition. For example, nonverbal refusal may mean that an elderly person turns his or her head when the caregiver offers food with a spoon during mealtime assistance. For the caregiver, humor, renarcissization, verbal refusal, and reformulation are at the top of both importance-related models.

Together with predictive tests that can be carried out by the nursing home team, these two measures can be very useful to develop protocols for successful care.

**Fig. 16:**
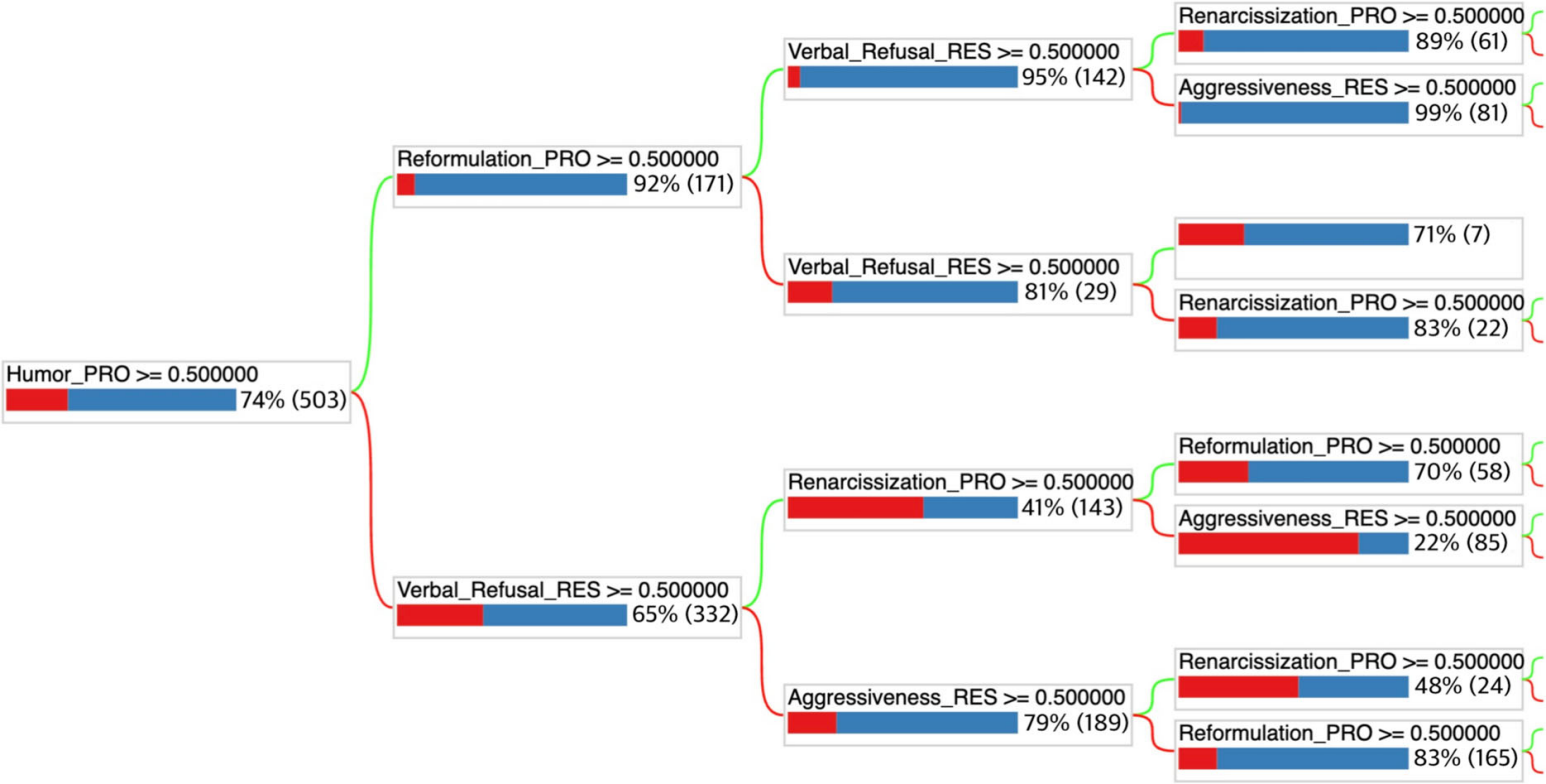
Decision tree with a selection of the top 5 features based on MDI. The tree is generated using the TensorFlow library. The impact of the splits on successful care can be observed, as well as the visualization of MDI importance

Indeed, if we consider the six most significant features—Renarcissization PRO, Nonverbal Refusal RES, Verbal Refusal RES, Humor PRO, Aggressiveness RES, and Reformulation PRO—a care protocol should prioritize these features as they enable the caregiver to make decisions quickly with a minimal set of features. Such a protocol can be based on experience or generated by AI. Once these features are identified, the model can be retrained using a selection of the most important features according to MDI/MDA. This results in a less effective decision tree, as shown in Fig. 16, which predicts successful care with only 81% accuracy but is smaller and therefore more practical for use without a tablet. The caregiver can also create a protocol of this type by selecting variables of interest, either following or not following the MDI/MDA values.

Figure 16 shows a portion of the decision tree. Two levels are missing, but the complete tree can be printed on a single sheet of paper. It illustrates the successful care based on different splits. At the root, without any division, the successful rate of the treatment is 74% for the 503 interactions. When the interactions are split based on the use of humor, two groups emerge. The first group, consisting of 171 interactions where humor is used, shows a 92% successful care. In the other group, where humor is not used, the successful care is only 65%. This figure highlights the subtle difference between MDI and PyXAI. Humor is a feature that allows for quick decision-making, while a feature like aggressiveness is crucial, as its absence seems almost essential for the treatment’s success. It is worth noting that printing decision trees on paper falls under a concept known as low-tech. This involves the use of simple, durable, and effective techniques, particularly in the healthcare field [77].

Note that, in both cases (measures), the carer’s experience has little to do with the model. However, this does not mean that these variables are not very important in determining the success or failure of care sessions. Rather, we examine the frequency with which they appear in a decision.

The critical importance of the elements of the interactions will be dealt with in the “PyXAI” section. The variables associated with interactions are therefore variables that are often present in decision trees and therefore need to be included in protocols and training.

**Fig. 17:**
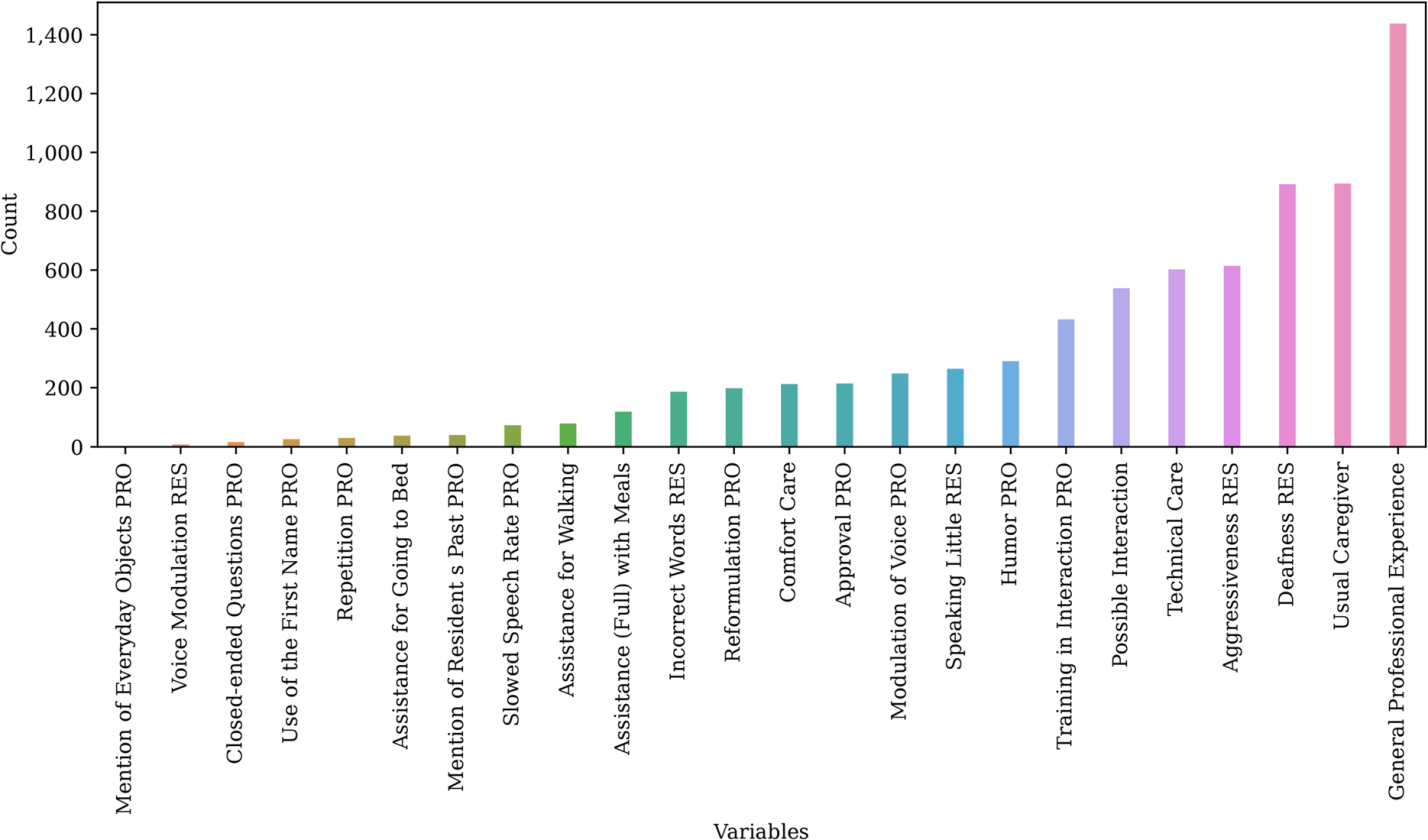
Number of occurrences of variables in minimal reasons (three minimal reasons computed per instance)

### PyXAI

In Fig. 17, we have calculated the number of occurrences of each variable and three minimum reasons per instance.

**Fig. 18:**
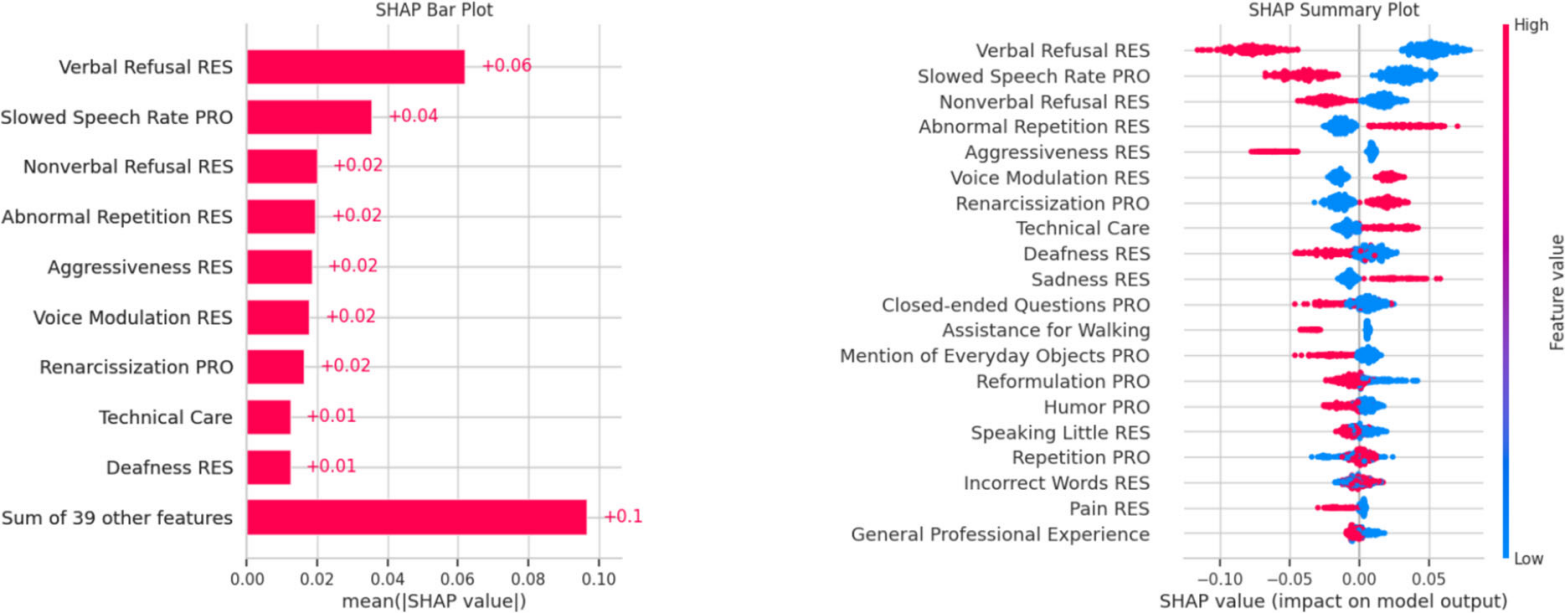
Contribution of variables to the hypothesis humor implies successful care. The graph on the left shows the features that contribute the most to this engagement. The graph on the right indicates the direction of the contribution: negative or positive

First, note that the two most frequent variables (on the right of the figure) in minimal reasons are contextual: they correspond to the caregiver’s general experience and local experience (habit) with the elderly.

The next two most frequent variables relate to the resident’s deafness and aggressiveness, demonstrating the impact of the resident on his or her care.

Possibility for interaction, training for interaction, and humor come next and seem to be less important variables, although we note that the first minimal reason resulting from the caregiver’s intervention and speech is humor.

### Causal forest: the impact of humor on care session

In this section, we specifically seek to establish a causal link between humor and successful care. We use a model called *causal forests* [10, 78] to understand such implications.

Given our previous findings, we might ask whether humor is a guarantee of successful care. To carry out our investigation, we decided to use the SHAP library and the causality explanation framework [47, 48].

Figure 18 shows the contribution of each variable to the model (left) and how they contribute (right).

**Fig. 19:**
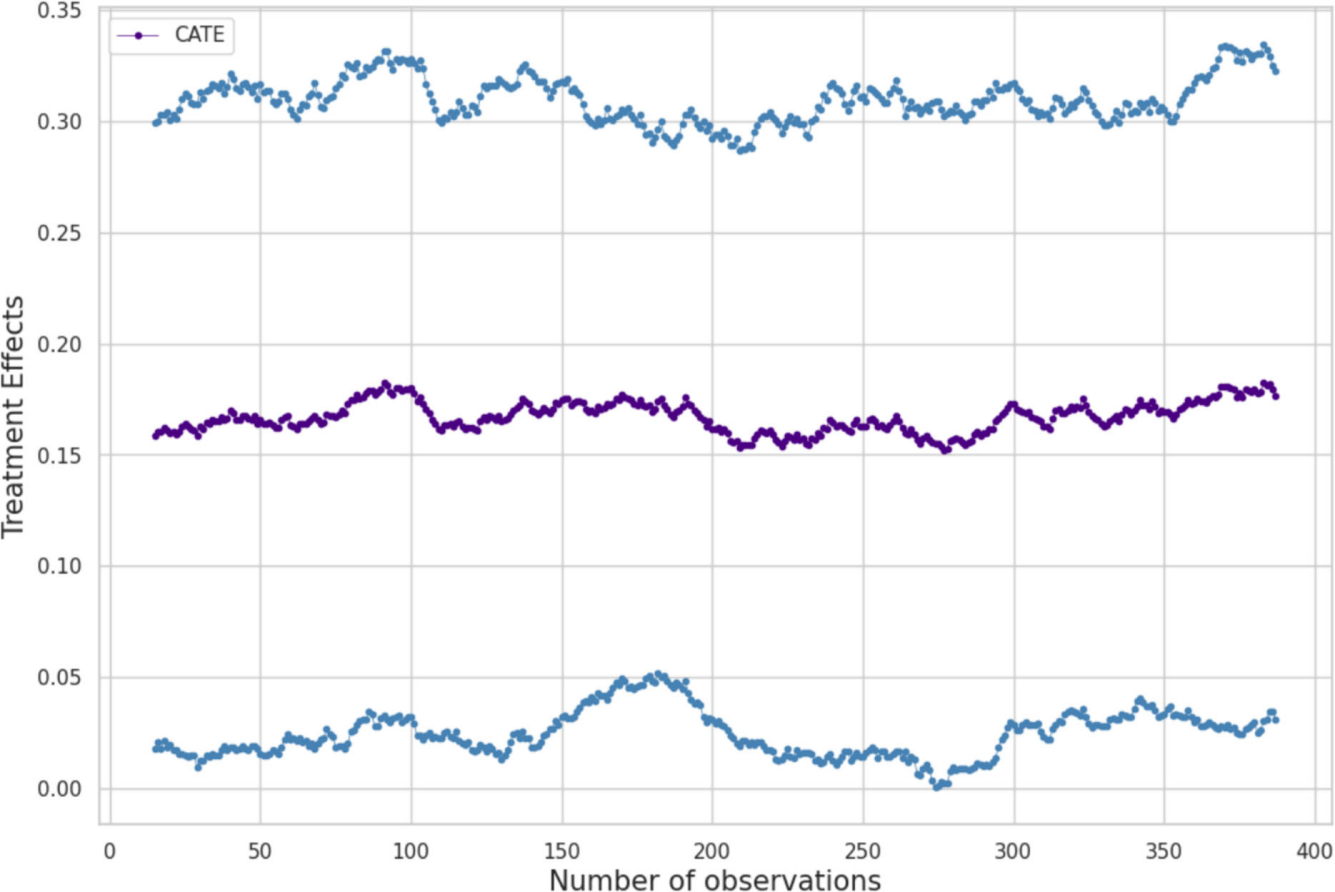
Impact of humor on Care. The CATE measure indicates whether an effect is genuine in the random forest algorithm. Here, the measure in purple is positive, indicating that the impact is real. The blue curves represent the confidence interval

Figure 19 shows a positive CATE for humor causality care. Interestingly, by isolating humor, we have found that it influences care, something we had already observed using other methods.

The figure on the right is complex. The red color indicates the presence of a feature, while blue indicates its absence. If a feature is in the positive section, it contributes positively to engagement; otherwise, it has a negative contribution. Notably, verbal and nonverbal refusals have negative contributions, as does the use of humor. Therefore, humor should be avoided in cases of verbal and nonverbal refusal. However, if no refusal is expressed by the resident, humor can be an asset. The case of aggressiveness is also interesting, as it shows that humor should be avoided when the resident is aggressive. Otherwise, it is neutral.

Additionally, when the caregiver slows their speech rate, humor is not effective. Finally, humor is effective when the patient exhibits abnormal repetitions, likely helping to diffuse the situation.

This demonstrates how humor can be highly effective, provided the caregiver pays close attention to the resident’s desires and their physical and mental state.

### RBM

**Fig. 20:**
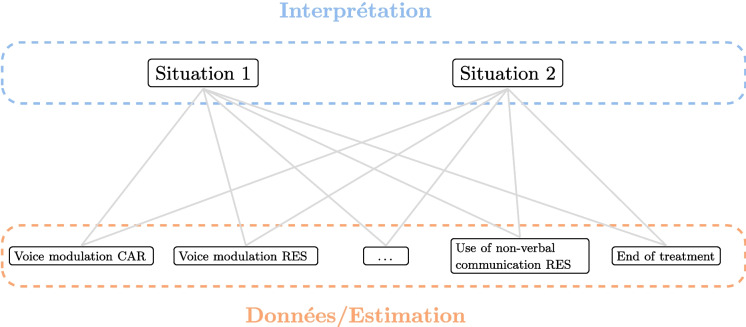
An RBM for analyzing care interactions. The neurons are binary variables that correspond to the features of our study

Having introduced it, we will now apply the contrastive divergence [6] algorithm to our dataset to train our RBM. Let us recall first that our dataset involves 50 binary variables. For this reason, we built an RBM with a visible layer consisting of 50 nodes, while the hidden layer consisted of only two nodes; see Fig. 20. The choice of only two nodes in the hidden layer was deliberate; the rationale being that one scenario was expected to be related to care success and the second to contextual factors. This was the vision of both the researchers and the carers, along a dialogue line. In fact, we have also tested with more than 2 scenarios, but, as imagined, the results were disappointing (probably because the correlations are scattered, making interpretation difficult).

**Table 4:** RBM results

Error per reconstruction	Accuracy	Precision	Recall	F1-score
20%	61.4%	69.8%	67.2%	68.4%

As expected, the RBM performs worse in predicting successful care compared to the random forest algorithm

This RBM was trained from our dataset. The general metrics obtained after training are shown in Table 4.

On the one hand, the quality of the training is considered to be quite good as the “error per reconstruction” is low. This is good news as it means that we can confidently use the RBM for simulating various scenarios: from incomplete input data, the RBM allows us to check and compute relevant probabilities of certain variables or partial scenarios. On the other hand, the values for accuracy, precision, recall, and F1-score are rather disappointing, which means that RBM is not very good at prediction (in our context). For example, overall precision is only 69.8%.

The strong completion capability allows for the estimation of features that the caregiver may not have completed, enabling the use of previous models in all cases.

The weights on the neurons are interpretable in terms of correlation.

Next, we have been interested in the correlations identified by the RBM.

By interpreting the correlation matrix, we have observed observed that:

- The first neural links show a correlation between:
  - The use of nonverbal language
  - The use of approval
  - The reformulation
  - The mention of everyday objects
  - The voice modulation as part of partial assistance with meals
- The second neural links show a correlation between:
  - A location of care not adapted
  - The resident’s nonverbal communication
  - The caregiver’s approval
  - Depersonalization
  - Humor
  - Voice modulation in both well-being and technical care

## Discussions

### RBM: insights into care dynamics

The analysis of the restricted Boltzmann machine (RBM) revealed two distinct care dynamics that, while sharing some correlation factors, highlight very different scenarios. The first neuron presents a resident capable of limited nonverbal communication and partial language use, requiring assistance with meals. In this case, the caregiver applies strategies such as affirmation, reformulation, voice modulation, and references to everyday elements. This approach aligns with common nursing home scenarios where caregivers use techniques to foster resident engagement, demonstrating how tailored communication can positively influence interactions.

However, the second neuron outlines a more problematic situation. Here, care is provided in unsuitable locations, such as hallways, and involves a resident with significant difficulties in both verbal and nonverbal communication. In this scenario, the caregiver’s use of humor, voice modulation, and depersonalizing language, such as collective pronouns (“we” instead of “you”) [71], mirrors “elderspeak.” This form of communication, often patronizing, can undermine the goals of care, especially in dementia contexts, where it may have a negative impact [29].

The aim of well-being care is to contribute to the restoration of self-esteem [53]. However, certain factors, especially those close to elderspeak, tend to resemble ageist behavior, which, on the contrary, leads to a negative self-perception [25].

These contrasting scenarios underline how essential it is to adapt care strategies based on the context and resident needs. While humor has been shown to reduce agitation and improve long-term well-being [45], its effectiveness is highly context-dependent.

### Feature analysis

The importance of language, active listening to residents, and the use of humor in interactions is also highlighted in methods other than those based on RBM. The significance of language for caregivers is notably marked by variables such as reformulation and renarcissization. Statistics on page 17 show an improvement in the success of care when caregivers adopt these strategies. The random forest algorithm highlights that these variables, and more generally, language-related variables, must be integrated into care protocols and caregiver training, as they help guide the interaction towards successful outcomes. The decision trees generated by AI offer insights into how language variables should be applied, producing simplified decision trees (see page 18).

The obtained results also indicate that features related to the resident’s willingness are very important. The features “Verbal Refusal” and “Nonverbal Refusal” appear significant in both descriptive statistics and random forest decisions. Therefore, the caregiver should consider the resident’s refusal as one of the key factors in the quality and success of the treatment. The decision tree in page 18 shows that interactions are rarely successful when the resident refuses the care. Hence, efforts should be made to obtain residents’ consent if possible, even if it means postponing the care. Furthermore, our study highlights the considerable effectiveness of humor in successful care. However, the causal forest method reveals that using humor in the context of care refusal is counterproductive. Emphasizing factors related to the acceptance of care by residents should be a priority in training and care protocols.

Our study also highlights a particular feature: the aggressiveness of the resident. This feature consistently shows very high measurements, regardless of the method used. It is important for successful care, for quick decision-making, and is often a crucial feature for achieving successful outcomes as exhibited by PyXAI. In other words, when the resident is aggressive, it is often not possible to achieve a successful care. It even constitutes a case in which it is recommended to avoid using humor. Therefore, it is important to consider ways to reduce the aggressiveness of residents, as this factor is associated with care failure in all scenarios.

Finally, the PyXAI library has demonstrated the indispensable role of experience in successful care. In many cases, an inexperienced caregiver will not be able to carry out the care successfully. This must be taken into account in the management of care in nursing homes. Too many inexperienced and poorly trained caregivers are responsible for elderly care in these facilities.

### Addressing data bias and generalizability

One notable limitation of this study is that the data were sourced from a single nursing home, which restricts the generalizability of the findings. This raises concerns about potential data bias, as the results may be influenced by factors specific to this institution.

Nevertheless, it is worth acknowledging that the resident profile of this facility aligns with national demographic trends in French nursing homes. This suggests some level of relevance, though not without limitations.

To enhance the generalizability of the results, future research should expand data collection to include a broader array of nursing homes, spanning both public and private sectors across different regions and countries. By widening the scope, researchers will be able to validate AI-driven insights in a variety of care environments, ensuring that recommendations are applicable to diverse settings. Incorporating facilities with varying standards of care, staff training methods, and resident populations would provide a more comprehensive understanding of care dynamics across the sector.

### Ethical considerations in the use of AI for elderly care

One of the major challenges in elderly care today is the lack of formal training for caregivers on how to interact with dependent elderly individuals. According to the Dress [2], there is a general deficiency in the training available to caregivers who work with this vulnerable population, leaving them unprepared to manage the complex interpersonal and communication needs of the elderly. This gap in training can lead to suboptimal care, particularly when it comes to communication, which is crucial for the emotional and psychological well-being of residents.

AI has the potential to help address this gap by emphasizing the importance of language and interpersonal communication in caregiving. By analyzing care interactions, AI systems can identify effective communication strategies and provide caregivers with concrete, data-driven recommendations. These insights could be used to develop training programs or tools aimed at improving caregivers’ abilities to engage with elderly residents in a compassionate and effective manner. This would bridge the gap in existing training programs and ensure that caregivers are better equipped to handle the complex communication needs of elderly individuals, particularly those with cognitive impairments or other dependencies.

However, while AI can play a valuable role in improving caregiver training and practice, it also raises important ethical concerns. The use of AI to analyze sensitive care interactions poses risks related to privacy, consent, and the potential over-reliance on automated systems. There is a danger that caregivers might become too dependent on AI, neglecting their own judgment and empathy, which are vital components of quality care. AI should complement the caregiver’s role, enhancing their capabilities rather than diminishing the human touch that is so important in elderly care.

Additionally, transparency is critical. Residents and their families need to be fully informed about how AI systems are being used and how their data are handled. Ensuring that all parties understand the role of AI in care interactions can help build trust and safeguard the dignity of the elderly individuals involved.

In light of these issues, it is essential to establish clear ethical guidelines for the use of AI in caregiving environments. AI should be positioned as a tool to support and enhance human care, not replace it. Proper policies must be developed to ensure that AI applications, especially those aimed at improving communication and interaction in caregiving, respect the rights, privacy, and autonomy of the elderly, while providing caregivers with the resources they need to offer better, more compassionate care.

### Optimizing care with AI: future directions

The findings from both RBM and PyXAI suggest that AI-driven insights can significantly enhance care practices, particularly by helping to fine-tune communication strategies. Building on these promising results, it is crucial to address the scope and generalizability of the current study. To do so, we plan to extend the data collection to include additional nursing homes across different regions and care environments. By expanding the dataset, we aim to validate the insights gained in a broader range of care contexts and ensure that the strategies we develop are applicable in diverse settings. This will also help mitigate the potential data bias that arises from working with a single facility.

Moreover, we are currently developing an application called COSIPAD (COmmunication et Soins Intelligents pour les Personnes gées Dépendantes). This application will be designed to help train caregivers on how to interact with dependent elderly residents. COSIPAD will offer personalized interaction strategies based on specific resident profiles and provide real-time feedback to caregivers. Additionally, COSIPAD could be used as a debriefing tool during team meetings to review care interventions and improve communication approaches, fostering a more collaborative care environment. By integrating AI-generated insights into COSIPAD, caregivers will be able to continuously refine their techniques, enhancing both the quality of care and the well-being of the residents.

Testing COSIPAD in real-world settings, such as nursing homes and home care environments, will provide invaluable feedback for refining these models and ensuring their practical applicability. Furthermore, this application could play a crucial role in standardizing best practices in care facilities and could be adapted for use in other care contexts, such as hospice care or at-home caregiving, where dynamics and resident needs vary.

### Integrating AI insights into caregiver training and policy

One of the key takeaways from this study is the importance of communication in caregiving, particularly the need to incorporate these insights into caregiver training programs. Current training tends to focus on technical skills, often neglecting the complex communication strategies necessary to navigate care interactions successfully. By integrating AI-derived insights, caregiver training can become more comprehensive, equipping future caregivers with the tools they need to manage both the technical and emotional aspects of elderly care.

Moreover, the findings should inform future policy standards. Policymakers could develop new regulations to ensure that communication skills are prioritized in caregiving training, while also establishing ethical guidelines for AI use in care environments. This would ensure that AI is used responsibly, supporting caregivers without compromising the human elements of care.

### Final thoughts and future perspectives

In summary, while the study provides valuable insights into the dynamics of caregiving through the use of AI models like RBM and PyXAI, its findings are limited by the fact that data were collected from a single nursing home. Future research should aim to validate these insights across a broader range of institutions to improve their generalizability. Despite this limitation, the study highlights the importance of communication in caregiving, particularly the role of humor, voice modulation, and other interaction techniques in enhancing care outcomes.

To address these challenges, we plan to extend the data collection to additional nursing homes and develop the COSIPAD application, which will serve as a training and feedback tool for caregivers. This application will not only help improve real-time care but also facilitate team debriefings and continuous improvement in caregiving practices. By integrating AI-driven insights into training and care environments, COSIPAD aims to enhance both the technical and emotional quality of care, fostering better interactions and improved outcomes for dependent elderly residents.

## Footnotes

1 Mainly the use of the first name and rarely in French, the equivalent of the diminutives honey and sweetie.

2 The MESHS junior valorization prize.

3 The Grammatica laboratory (UR4521) and the CRIL laboratory (UMR 8188). These labs are also collaborating within the MAIA (Mastering AI Applications) project, which is funded by the French government with a budget of 11 million euros.

4 Sadness, abnormal repetition, refusal, etc.

5 Approval, renarcissism, etc.

6 Diaper, bib, potty.

7 Positive and negative answers collected for all decision trees.

8 By Jeremybeauchamp - Personal work, CC BY-SA 4.0, Wikipedia.
